# The Role of Mitogen-Activated Protein Kinase-Activated Protein Kinases (MAPKAPKs) in Inflammation

**DOI:** 10.3390/genes4020101

**Published:** 2013-03-26

**Authors:** Ugo Moens, Sergiy Kostenko, Baldur Sveinbjørnsson

**Affiliations:** Molecular Inflammation Research Group, Department of Medical Biology, Faculty of Health Sciences, University of Tromsø, NO-9037 Tromsø, Norway; E-Mails: sergiy.kostenko@uit.no (S.K.); baldur.sveinbjornsson@uit.no (B.S.)

**Keywords:** RSK, MSK, MNK, MK2, MK3, MK5, NF-κB, STAT3

## Abstract

Mitogen-activated protein kinase (MAPK) pathways are implicated in several cellular processes including proliferation, differentiation, apoptosis, cell survival, cell motility, metabolism, stress response and inflammation. MAPK pathways transmit and convert a plethora of extracellular signals by three consecutive phosphorylation events involving a MAPK kinase kinase, a MAPK kinase, and a MAPK. In turn MAPKs phosphorylate substrates, including other protein kinases referred to as MAPK-activated protein kinases (MAPKAPKs). Eleven mammalian MAPKAPKs have been identified: ribosomal-S6-kinases (RSK1-4), mitogen- and stress-activated kinases (MSK1-2), MAPK-interacting kinases (MNK1-2), MAPKAPK-2 (MK2), MAPKAPK-3 (MK3), and MAPKAPK-5 (MK5). The role of these MAPKAPKs in inflammation will be reviewed.

## 1. Introduction

Inflammation is essential for tissue response to injury, trauma or infection. The healing process is initiated by specialized cells and the release of inflammatory modulators. The inflammatory response starts with the biosynthesis of pro-inflammatory cytokines such as tumor necrosis factor-α (TNF-α) and interleukin-1β (IL-1β) both of which promote tissue repair and regeneration. However, complex feedback mechanisms that carefully regulate the magnitude and duration of the inflammatory response are required because unrestrained inflammatory responses can cause tissue damage and be the underlying *modus operandi* of chronic inflammatory diseases [1]. The production of inflammatory modulators is controlled by several signaling pathways, including the nuclear factor-kappa B (NF-κB), the Janus kinase/signal transducer and activator of transcription (JAK-STAT) and the mitogen-activated protein kinase (MAPK) pathways (for excellent recent reviews see [2,3,4,5]). The present review focuses specifically on the roles of the MAPK-activated proteins (MAPKAPK) in inflammatory processes, but will first briefly describe the composition and functions of the major human MAPK pathways.

**Figure 1:**
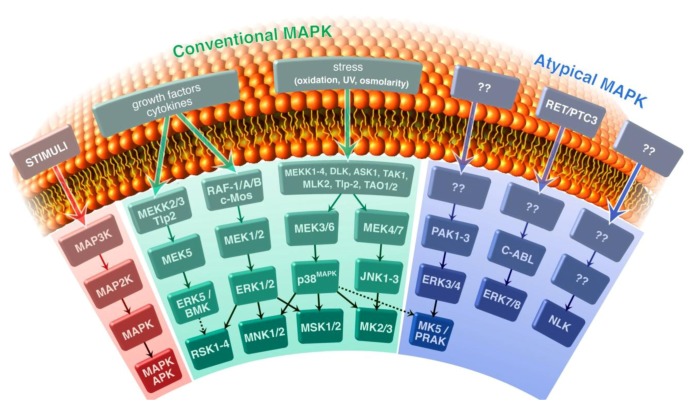
Schematic presentation of the mammalian mitogen-activated protein kinase(MAPK) pathways. The conventional MAPK pathways, represented by MAPK/extracellular signal regulated kinase kinase (MEK), extracellular signal-regulated kinases (ERK), c-Jun N-terminal kinases (JNK), p38^MAPK^, and MEK5/ERK5 and the atypical MAPK pathways, including ERK3, ERK4, ERK7, ERK8, and Nemo-like kinase (NLK) are shown. The typical MAPK pathways consist of a module of three kinases that subsequently phosphorylate and activate each other: MAPK kinase kinase (MAP3K) phosphorylates MAPK kinase (MAP2K), which in turn phosphorylates MAPK. The MAPKs ERK1, ERK2 and ERK5 are typically activated by growth factors, while p38MAPK and JNK are predominantly induced by a variety of environmental stresses. JNK can also be activated by mitogens and pro-survival stimuli. Downstream of MAPK are substrates including other protein kinases referred to as MAPK-activated protein kinases (MAPKAPK). The MAPKAPK MK5/PRAK is target for the conventional p38 MAPK and the atypical ERK3/4 MAPKs.

In mammalian cells, seven different MAPK pathways have been described: extracellular signal-regulated kinases 1 and 2 (ERK1/2), JNK, p38^MAPK^, ERK5, ERK3/4, ERK7/8, and the Nemo-like kinase (NLK; Figure 1). They can be divided into four conventional and three atypical MAPK pathways. The typical MAPK pathways consist of a relay of three phosphorylation events in which a MAPK kinase kinase (MAPKKK or MAP3K) phosphorylates a MAPK kinase (MAPKK or MAP2K), which in turn phosphorylates a MAPK. This three tiered structure seems absent in the atypical MAPK pathways ERK3/4, ERK7/8 and NLK (for recent reviews see [3,6]). The typical MAPKs and the atypical ERK3/4 can phosphorylate other protein kinases designated as MAPK-activated protein kinases (MAPKAPK). The MAPKAPK family comprises the ribosomal-S6-kinases (RSK1-4), the MAPK-interacting kinases (MNK1 and 2), the mitogen- and stress-activated kinases (MSK1 and 2), MAPKAPK-2 (MK2) and MAPKAPK-3 (MK3), and MAPKAPK-5 (MK5) [7,8,9,10,11,12].

RSKs are downstream targets of the MAPKs ERK1, ERK2 and ERK5 and regulate cell growth, cell proliferation, cell survival, transcriptional and translational regulation, and cell motility [3,6,7,10]. MNK1 and MNK2 were originally identified by screening for ERK1/2 substrates, but it was later shown that they are activated *in vivo* by both ERK1/2 and p38^MAPK^ [3,6,9]. The identification of substrates proposes the possible involvement of MNK in transcriptional and translational regulation, inflammatory responses, proliferation and survival [3,6,9]. MSK1 and MSK2 (or RSKB), downstream targets of ERK1, ERK2, and p38^MAPK^, play a versatile role in transcriptional and translational regulation, and affect inflammatory responses and neuronal processes [3,6,8]. MK2 and MK3 possess 75% sequence identity and are directly activated by p38^MAPK^ [11,13]. MK2 knockout mice are fertile, viable, of normal size and depict no specific behavioral defects, yet display increased stress resistance and survive LPS-induced endotoxic shock [14]. MK3 deficient mice are viable, fertile and possess an apparent wild-type phenotype under normal conditions, but in contrast to MK2, they do not display altered cytokine production in response to lipopolysaccharide (LPS) [15]. However, studies in a MK2-deficient background indicated that MK3 can compensate for the loss of MK2 [15]. Together with sequence and domain analysis information, these data suggest that from a functional perspective MK2 and MK3 are closely related enzymes. MK5 is most closely related to MK2 and MK3, but seems to be less functionally related [13]. MK5 knockout mice on a mixed 129xC57/B6 genetic background are viable, while for reasons unknown they show embryonic lethality on a C57BL/6 genetic background. MK5 is activated by the atypical MAPKs ERK3and ERK4. MK5 can also be activated by p38^MAPK^ [12,16]. MK5 possesses anti- and pro-tumorigenic properties, but may also be involved in neurosecretion [17,18]. 

## 2. RSK and Inflammation

The heterodimeric transcription factor nuclear factor-kappa B (NF-κB) is a key regulator of a broad range of genes involved in inflammatory processes [19,20]. NF-κB, which is typically composed of the p65 (RelA) and p50 (NF-κB1) subunits, resides as an inactive complex with inhibitory IκB proteins in the cytoplasm and can be activated by the ubiquitin-dependent degradation of IκB in the 26S proteasome. Phosphorylation of IκB is pivotal for the induction of polyubiquitination and proteasomal degradation of IκB [21]. IκB kinases (IKK), as well as other protein kinases can phosphorylate IκB. One of these kinases is RSK, which phosphorylates IκBα at Ser-32, thereby eliciting activation of NF-κB [22,23]. Activation of NF-κB can also be achieved by phosphorylating and activating IKK, which will subsequently phosphorylate IκB. Additionally, angiotensin II can be implicated in chronic inflammatory reactions because angiotensin II can stimulate expression of the proinflammatory chemokine IL-6 and attenuates IL-1β induced activation of NF-κB [24,25,26]. Several studies have unveiled a role for RSK in angiotensin II-induced activation of NF-κB. Doyen and Servant showed that angiotensin II activates NF-κB by phosphorylating IKKβ on Ser-177 and Ser-181, resulting in the subsequent phosphorylation and degradation of IκBα. Phosphorylation of IKKβ was partially mediated by RSKs because the RSK1-4 inhibitor BI-D1870, as well as siRNA against RSK1, -2, and -3 impaired IKKβ phosphorylation and IL-6 mRNA production after angiotensin II treatment [27]. How RSK accomplishes phosphorylation of IKKβ remains elusive because despite a physical interaction between RSK and IKKβ, RSK does not directly phosphorylate I kappa B kinase (IKKβ) [28]. Two studies by Zhang and colleagues demonstrate that angiotensin II induces phosphorylation of the NF-κB p65 subunit at Ser-536, causing increased binding of p65 to target sequences. Angiotensin II-triggered phosphorylation of p65 at Ser-536 is mediated by RSK1/2 and by IκB kinase in vascular smooth muscle cells [29,30]. A role for RSK in phosphorylation of p65 at Ser-536 is supported by the finding that RSK1 can also mediate p53-induced p65 phosphorylation at this residue and this phosphorylation reduces p65’s affinity for IκBα [31]. 

RSK-mediated phosphorylation of the NF-κB p65 subunit is also implicated in response to the activation of proteinase-activated receptors (PARs). PARs are G-protein-coupled receptors activated through proteolysis of the receptor rather than ligand binding and are involved in intestinal inflammation [32]. IL-8 plays an important role in the pathogenesis of inflammation of the gut and the activation of PAR stimulates IL-8 expression [33,34]. Wang and co-workers showed that PAR activation in HT-29 human colonic epithelial cells induces phosphorylation of the NF-κB p65 subunit at Ser-276 and Ser-536 and this coincided with enhanced transcriptional activation of the *il-8* gene [35]. They showed that phosphorylation of Ser-276 is mediated by RSK-1/2, but not MSK-1, while phosphorylation of Ser-536 occurs in an ERK1/2-independent manner. PAR activation also enhanced recruitment of histone acetyltransferase p300 to the NF-κB p50 subunit, while reducing the association of this unit with histone deacetyltransferase 2. Activation of PAR also stimulates expression of IL-6 and prostaglandin E_2_ (PGE_2_), but it remains to be determined as to whether RSK is involved in this process [34]. In conclusion, RSK may stimulate transcription of genes encoding inflammatory mediators through activation of NF-κB by mediating phosphorylation of IKK or the p65 subunit at Ser-276 or Ser-536 depending on the upstream stimulus. 

RSK participates in the expression of cyclooxygenase 2 (COX2) in response to lipopolysaccharide (LPS) by an NF-κB-independent mechanism [36]. LPS, a component of the cell wall of Gram-negative bacteria, activates macrophages to produce pro-inflammatory cytokines such TNF-α and IL-1β, and secondary mediators such as prostaglandins. The rate-limiting step in prostaglandin synthesis is catalyzed by cyclooxygenase COX-2, which is expressed at very low levels, but is strongly induced by proinflammatory stimuli such as LPS and COX-2 [37,38,39]. Eliopoulos and associates showed that LPS-induced secretion of PGE_2_ in macrophages correlates with increased transcription of the *cox-2* gene, but also stabilization of COX-2 mRNA. Enhanced transcription is mediated by transcriptional activation of CREB (cAMP response element-binding protein)/ATF-1 (activating transcription factor 1) through a signaling pathway engaging Tlp2 Ser/Thr kinase, and the MEK1/2-ERK1/2 pathway and the MAPKAPKs RSK and MSK [36]. RSK and MSK may exert additional effects on transcription though phosphorylation of the chromosomal proteins histone H3, HMGN1 and histone acetyltransferase CBP (CREB-binding protein) [6]. The mechanism by which the Tlp2/ERK pathway regulates COX-2 mRNA stability remains to be solved. Knowing the established roles of COX-2 and PGE_2_ in inflammation, these findings imply that RSK and MSK play an important role in the response to the inflammatory signal LPS.

RSKs seems to be exclusively activated by ERK1/2, however in dendritic cells an ERK1/2-independent activation of RSK was identified. This ERK1/2-independent activation of RSK is achieved through MK2/3-mediated phosphorylation of RSK at Ser-386. The cell-specificity of this dual mode of RSK activation may be explained by a cell-specific scaffolding protein that permits RSK-MK2/3 interaction. The MK2/3-dependent activation of ERK1/2 allows dendritic cells to engage several pathways for stimuli that are weak ERK1/2 activators to obtain strong activation of ERK1/2 after all [40]. The biological relevance of this cross-talk in inflammatory responses is not known. 

## 3. MSK and Inflammation

### 3.1. MSK and Expression of Inflammatory Regulators

MSK1 and MSK2 are MAPKAPKs that are targets of the MAPKs ERK1/2 and p38^MAPK^. These MAPKs can directly phosphorylate MSK1 at Ser-360, Thr-581 and Thr-700, while autophosphorylation occurs at Ser-212, Ser-376, Ser-381, Ser-750, Ser-752 and Ser-758 (reviewed in [8]). Recently, it was shown that mixed lineage kinase-related kinase-β (MRK-β) can phosphorylate additional sites, including Ser-7, Ser-8, Thr-216, Tyr-375, Ser-436, Ser-438, Thr-740 and Thr-749 [41]. MSK1 has been suggested to play a role in inflammation because of its apparent property to mediate phosphorylation of the NF-κB p65 subunit at Ser-276 upon IL-1β or TNF-α treatment [42,43,44,45]. This post-translational modification stimulates recruitment of the histone acetyl transferases CREB-binding protein (CBP) and p300. Moreover, CBP and p300 are also able to acetylate the NF-κB p65 subunit itself. These processes result in increased transcriptional activity of NF-κB [46]. However, MSK-mediated phosphorylation of p65 at Ser-276 *in vivo* is highly questionable because the phosphoSer-276 antibodies used in these studies are not specific and detect phosphoproteins of inappropriate molecular mass, and are not antigenically related to p65 [47]. Despite this, MSK1 may be implicated in inflammatory processes because this kinase can be activated by inflammatory regulators and pathways. TNF-α induces phosphorylation of MSK-1 at Ser-360, Ser-376, and Thr-581 in airway smooth muscle cells. The unspecific NF-κB inhibitor dimethylfumarate reduced TNF-α-triggered MSK1 Ser-376 autophosphorylation, but not Ser-360 and Thr-581 phosphorylation. Dimethylfumarate also prevented IL-1β-provoked MSK1/2 phosphorylation in human keratinocytes [48]. The inhibitory effect of dimethylfumarate on TNF-α- and IL-1β-induced phosphorylation of MSK1 was independent of the p38^MAPK^ and ERK pathways because no changes in ERK1/2 and p38^MAPK^ activation were observed in the presence of the inhibitor [48,49]. This is confirmed by the finding that the phosphorylation of ERK1/2 and p38^MAPK^ phosphoacceptors sites Ser-360 and Thr-581 were unaffected in the presence of dimethylfumarate. The use of the unspecific NF-κB inhibitor may suggest that NF-κB can mediate activation of MSK in response to inflammatory cytokines such as TNF-α and IL-1β, but the exact mechanism for NF-κB-mediated phosphorylation of MSK in response to TNF-α and IL-1β remains unknown. This stimulatory NF-κB: MSK loop may improve inflammatory responses. A role for MSK1 in cutaneous inflammation in response ultraviolet B (UVB) light is suggested by the findings of Terazawa and co-workers who found that UVB irradiation stimulated secretion of PGE_2_ and IL-8 in keratinocytes, while H-89, an inhibitor of MSK (see Section 8.2) attenuated this secretion [50]. 

Toll-like receptors (TLRs) are major components of the innate immune system that function in promoting inflammation, although TLRs can also activate anti-inflammatory signals critical in preventing excessive inflammation [51,52]. MSK1 operates downstream of several TLRs because lipoteichoc (TLR2), LPS (TLR4), tripalmitoyl cysteinyl seryl tetralysine lipopeptide (TLR1/2/6), and CpG (TLR9) can all activate MSK1 [53,54]. The same authors showed that MSK1/2-deficient bone marrow-derived macrophages (BMDM) secrete more pro-inflammatory cytokines IL-6, IL-12, and TNF-α than wild-type BMDM upon LPS exposure, thereby suggesting that MSK1/2 suppresses expression of these cytokines. LPS activates the TLR4-p38^MAPK^-ERK1/2 pathway, resulting in MSK1/2 activation and subsequent phosphorylation of the transcription factors CREB and ATF-1 at Ser-133 and Ser-63, respectively, which will increase the expression of the MAPK phosphatase DUSP1 (dual specificity phosphatase 1) and the anti-inflammatory cytokine IL-10. DUSP1 will inhibit p38^MAPK^ and hence further activation not only of its substrate MSK1/2 but also MK2/3, while IL-10 inhibits the expression of IL-6, IL-12 and partially that of TNF-α via STAT3 [53]. A similar mechanism seems to be operational for LPS-induced expression of the inflammatory proteins endothelin-1 and plasminogen activator inhibitor-2, an inhibitor of IL-1β secretion in BMDM. Kim and colleagues demonstrated that expression of IL-10, DUSP1 and plasminogen activator inhibitor-2 was impaired in MSK1/2-deficient BMDM after LPS stimulation. Therefore MSK1/2 may be implicated in damping the inflammatory response by modulating the expression of DUSP1 and plasminogen activator inhibitor-2 [55].

MSK1/2 are also involved in the expression of the anti-inflammatory cytokine IL-1ra (IL-1 receptor antagonist), which is able to bind but not activate the IL-1 receptor [56]. The authors showed that exposure of BMDM to the TLR4 agonist LPS resulted in elevated IL-1ra mRNA and protein levels while a significant reduction in IL-1ra expression was observed in MSK1/2 deficient BMDM compared to wild-type macrophages in response to LPS. Similar results were obtained using TLR2 or TLR9 agonists [56]. The exact mechanism by which MSK1/2 stimulates IL-1ra transcription is not known, although both IL-10-dependent and –independent pathways seem to be implicated [56]. MSK1/2 regulate IL-10 production [50] whereas IL-10 has a stimulatory effect on IL-1ra transcription [57]. However, IL-10 alone could not restore LPS-induced IL-1ra expression in MSK1/2 knockout macrophages to the levels measured in wild-type cells, whiles the triple knockout of IL-10 and MSK1/2 resulted in a greater decrease in IL-1ra mRNA induction than IL-10 knockout alone [56].

Asthma is a common chronic inflammatory disease of the airways and IL-11 and IL17F are considered as critical cytokines in the pathogenesis of airway inflammation [58,59,60,61]. By using protein kinase-specific inhibitors, phospho-specific antibodies and siRNA interference, Kawaguchi and colleagues demonstrated that IL-17F induces IL-11 expression by the Raf1-MEK-ERK1/2-MSK1-CREB signaling pathway in bronchial epithelial cells [62]. Insulin-like growth factor-I (IGF-I) is a growth factor involved in the pathogenesis of airway inflammation [63]. The Kawaguchi group reported that in bronchial epithelial cells, IL17F triggers IGF-I expression via MSK1- and RSK-mediated activation of CREB [64]. These results suggest that MSK1 and RSK are involved in the pathogenesis of asthma via IL-17F induction of IL-11 and IGF-I.

The JAK/STAT pathway plays an important role in inflammatory responses and one of its members, the transcription factor STAT3, is rapidly activated by various cytokines, a process that requires phosphorylation of serine residue 727 [5]. UVA irradiation and erythropoietin induce phosphorylation of STAT3 at Ser-727 by MSK1, but it is not known whether MSK1 is involved in cytokine-triggered phosphorylation of STAT3 [65,66]. A possible connection between MSK1, STAT3 and inflammatory responses remains to be established.

Finally, several kinases downstream of p38^MAPK^, including MSK, MNK, and MK5, but neither MK2 nor MK3 can phosphorylate cytoplasmic phospholipase A2 (cPLA_2_) at Ser-727 *in vitro*. This enzyme cleaves membrane lipids to release arachidonic acid, the COX-2 target and phosphorylation of cPLA_2_ stimulates its enzyme activity [67]. It remains to be determined whether MSK is a genuine cPLA_2_ kinase while the possible functional implication of MSK-mediated phosphorylation of cPLA_2_ in inflammation has not been established. 

### 3.2. MSK and *In Vivo* Models of Inflammation

The importance of MSK1/2 inflammation *in vivo* has been confirmed in three different animal models [53]. Intraperitoneal injection of LPS induced higher serum levels of TNF-α, IL-6 and IL-12 and lower amounts of IL-10 in MSK1/2 double knockout mice compared to wild-type mice, thus confirming the role of MSK1/2 in the regulation of pro- and anti-inflammatory cytokine production observed in BMDM. MSK1/2 double knockout mice in which the cecum was ligated and punctured (a murine model of human septic shock) produced significantly less IL-10 than wild-type controls, while IL-6, IL-10 and TNF-α production was not substantially affected. In an acute toxic contact eczema model in which local inflammation was induced by phorbol ester, MSK1/2 deficiency failed to resolve inflammation because myeloperoxidase activity in extracts of ear biopsies and cellular infiltration in the dermis were much higher in double-knockout mice compared to wild-type animals 72 and 96 hrs after phorbol ester treatment [53]. 

Using an oxazolone-induced allergic contact dermatitis model in mice, Bertelsen and colleagues monitored inflammatory responses in wild-type and MSK1/2 double knockout mice. They found increased signs of inflammation in the MSK1/2 deficient mice compared to wild-type littermates, which was manifested by increased ear thickness and inflammatory histological changes, elevated infiltration of neutrophils (higher myeloperoxidase activity) in the skin, higher TNF-α, IL-1β, and IL-6 mRNA and protein levels, more pronounced infiltration of CD4+ and CD8+ T cells, and augmented thymus and activation regulation chemokine (TARC, a chemokine implicated in skin inflammation) transcript levels [68]. Accordingly, phorbol-ester treatment also resulted in a sustained and elevated inflammatory response in the skin of MSK1/2 deficient mice compared with wild-type mice, suggesting that MSK1/2 are required to reduce inflammation in chronic inflammatory skin diseases [69]. The mechanism by which lack of MSK1/2 results in elevated inflammatory responses after oxazolone treatment remains unknown because no differences in IL-10 and DUSP1 expression were measured between wild-type and double knockout mice [68]. IL-10 (by inhibiting expression of the pro-inflammatory cytokine IL-6) and DUSP1 (by inactivating p38^MAPK^) both exert anti-inflammatory effects [53]. Despite the more pronounced inflammation in MSK1/2 deficient mice compared to wild-type mice in response to oxazolone, the levels of IL-6 and DUSP1 were similar in both groups of animals [68]. Hence, the anti-inflammatory role of MSK1/2 in oxazolone treated mice seems to be DUSP1- and IL-10- independent. 

In conclusion, because MSK1/2 target both pro- and anti-inflammatory genes, these protein kinases operate on a tight equilibrium of positive and negative feedback mechanisms in the immune system [70].

## 4. MNK and Inflammation

Another enzyme that contributes to prostaglandin synthesis is cPLA_2_ whose expression is induced by cytokines such as IFN-α and IL-1. [71,72]. MNK1 phosphorylates cPLA_2_ at Ser-727 *in vitro* and overexpression of non-phorylatable cPLA_2_ S727A mutant or of inactive MNK1 impairs arachidonate release [67]. This study indicates that MNK1 phosphorylates cPLA_2_*in vivo*, which then activates the enzymatic activity of cPLA2. Studies with MNK-specific inhibitors, siRNA-mediated depletion of MNK or in MNK deficient cells/animals may help in establishing the *in vivo* role of MNK1 in regulating cPLA_2_. MNK1 also regulates the production of several pro-inflammatory cytokines, including IL-6 and TNF-α [73,74,75]. MNK phosphorylates the AU-rich element (ARE) binding protein heterogeneous nuclear ribonucleoprotein A1 (hnRNP A1) and this causes decreased binding of hnRNP A1 to the TNF-α mRNA which may stabilize and/or stimulate translation of TNF-α mRNA [73,76]. Interestingly, MNK inhibition stimulates LPS-induced anti-inflammatory cytokine IL-10 production in macrophages [75]. This observation suggests that an anti-inflammatory response (*i.e*., IL-10 production) is not prevented by MNK inhibition, while proinflammatory responses (*i.e.*, TNF-α and IL-6 production) are reduced. This makes MNK inhibitors promising therapeutic agents in inflammatory diseases in which bacterial products and proinflammatory cytokines play a critical role in their pathogenesis [75]. 

## 5. MK2 and Inflammation

### 5.1. MK2 and Regulation of Inflammatory Mediators

Previous experiments have shown that p38^MAPK^ inhibitors impair production of pro-inflammatory cytokines although a possible role of the p38^MAPK^ substrate MK2 in inflammatory processes is unknown [77]. The assumption that MK2 might be implicated in inflammation was reinforced by the phenotypic changes observed in MK2 knockout mice. Studies with MK2-deficient mice have revealed that these animals survive doses of LPS that are lethal for wild-type littermates and display a significant reduction in biosynthesis of TNF-α, IFN-γ, IL-1β, IL-6, and IL-10 compared with wild-type mice [14]. Furthermore, several studies with different cell types obtained from MK2^−/−^ mice confirmed the role of MK2 in the production of these inflammatory modulators [14,78,79,80,81,82,83]. 

MK2 employs several complex mechanisms to regulate the expression of inflammation modulators and inflammatory molecules may activate MK2 to help guarantee a positive feedback. Indeed, IL-1β was shown to activate MK2 in human keratinocytes and human umbilical vein endothelial cells, thus suggesting a positive feedback mechanism between MK2 and IL-1β [84,85]. MK2 may further enhance the stability of selected transcripts through enhancement of the activity of RNA stabilizing factors such as hnRNP A0 or through inhibiting the activity of mRNA destabilizing protein such as tristetraprolin [86,87,88]. MK2 can also affect the expression of inflammatory modulators at the level of transcription. Examples and the molecular mechanisms by which MK2 controls the expression of pro- and anti-inflammatory molecules are discussed in this section.

MK2-regulated TNF-α biosynthesis is complex and several ARE binding proteins such as human antigen R (HuR), tristetraprolin (TTP) and hnRNP A0 play a pivotal role ([73,78,86,87,89,90] and reviewed in [91]). HuR may stabilize TNF-α mRNA by binding in a MK2-dependent manner while binding of unphosphorylated TTP to TNF-α mRNA induces degradation of the transcript [87,90,92]. MK2 phosphorylates TTP at Ser-52 and Ser-178 and phosphorylated TTP: TNF-α mRNA complexes are sequestered by 14-3-3 proteins [90]. Moreover, phospho-TTP is unable to recruit deadenylases, resulting in target mRNA stabilization [88,93]. Another result of TTP phosphorylation by MK2 is stabilization and increased translation of TTP mRNA. MK3 also contributes to TTP mRNA stabilization. Finally, MK2 and MK3 stimulate *de novo* transcription of TTP through phosphorylation of the transcription factor serum response factor [94,95]. The dual action of MK2/3 on TTP phosphorylation and transcription may represent a default mechanism in limiting inflammation [91]. Moreover, phosphorylation of the RNA stabilizing factor protein hnRNP A0 at Ser-84 by MK2 increases the binding to and stabilization of TNF-α mRNA [86]. MK2 deficient mice cannot phosphorylate TTP and hnRNP A0, hence unphosphorylated TTP will stimulate the degradation of TNF-α mRNA, while unphosphorylated hnRNP A0 cannot prevent TNF-α mRNA decay. This explains the reduced levels of TNF-α in LPS-treated MK2^−/−^ mice compared with LPS-treated wild-type animals. The expression of IL-8, which is released from different types of cells in response to the proinflammatory cytokine IL-1, is partially controlled by MK2. A constitutive active MK2, though not a kinase inactive mutant, stabilizes IL-8 mRNA, suggesting that MK2-mediated phosphorylation of the RNA destabilizing protein TTP impairs the degradation of IL-8 mRNA [96]. IL-8 mRNA stabilization is further regulated by the destabilizing protein KSRP (KH-type splicing protein). IL-1 treatment impaired the interaction of KSRP with IL-8 mRNA in a p38^MAPK^-dependent, but MK2-independent manner [97]. Thus the p38^MAPK^ pathway modulates IL-8 mRNA stability through a dual mechanism: One involves KSRP and is MK2-independent, while the other requires MK2-mediated phosphorylation of TTP. More than 100 TTP-regulated mRNAs, including the transcripts for IL-2, IL-6, IL-10 and IFN-γ have been identified, so MK2 may also modulate their stability [98,99,100]. Taken together, MK2-dependent stabilization of mRNA encoding inflammatory modulators is an important function for MK2 in inflammation. 

MK2 not only affects the expression of inflammatory modulators at the post-transcriptional level, but may also stimulate transcription of their genes by modifying the action of transcriptional activators or repressors. Heat-shock factor 1 (HSF1) can repress the transcription of proinflammatory IL-1β cytokine [101]. MK2 phosphorylates HSF1 on Ser-121, which impedes the binding of HSF1 to cognate elements in promoters [102]. Although not addressed by the authors, MK2-mediated phosphorylation of HSF1 may therefore release the transcriptional repression of the *il-1β* gene.

A third mechanism by which MK2 can interfere with inflammatory responses is by obstructing signaling pathways. IL-6 is a cytokine that can exert pro- and anti-inflammatory activities [103]. MK2 restricts IL-6 signaling induced by the pro-inflammatory cytokines TNF-α and IL-1β and stress by phosphorylating the common gp130 subunit of the IL-6 receptor at Ser-782. This posttranslational modification results in increased internalization and degradation of the receptor, and subsequently declined STAT3 activation and reduced stimulation of STAT3-mediated gene transcription [104]. IL-1β- or stress-induced gp130 Ser-782 phosphorylation was significantly reduced in MK2^−/−^ cells, but not in MK5-deficient cells. This residual phosphorylation may therefore be attributed to MK3, a MAPKAPK structurally related to MK2 (see below). A recent study demonstrated that MK2 can regulate NF-κB activity [105]. Protein inhibitor of activated STAT1 (PIAS1) can interact with NF-κB and repress its transcription activity [106]. Heo and co-workers reported that MK2 can phosphorylate PIAS1 at Ser-522 *in vitro* and *in vivo*. Depletion of MK2 prevented TNF-α triggered PIAS1 phosphorylation at Ser-522, and the PIAS1 mutant in which Ser522 was substituted by non-phosphorylatable Ala (PIAS1 S522A) was unable to repress TNF-α-induced NF-κB transcriptional activity. Overexpression of wild-type, but not mutant PIAS1 S522A, inhibited TNF-α-induced expression of inflammatory genes in HUV-EC, while PIAS1 S522A could not inhibit TNF-α-provoked endothelial inflammation in a mouse skin transplant model [105]. In conclusion, aberrant activity of MK2 perturbs the balanced regulation of the NF-κB pathway and may therefore contribute to inflammation. A role for MK2 and MK3 in controlling the NF-κB pathway will be discussed in Section 6.

### 5.2. Phosphorylated HSP27 and Inflammation

MK2 may affect inflammatory responses through one of its substrates, heat-shock protein HSP27, which is implicated in inflammation. However, the exact mechanisms are incompletely understood, although in some cases the phosphorylation status of HSP27 seems to be of importance. This protein can be phosphorylated on serine residues 15, 78 and 82 by MK2, MK3 and MK5, although with different stoichiometry and kinetics and under different conditions [107,108,109,110]. Some examples of how phosphorylated HSP27 is implicated in inflammation are discussed in here, but evidence for MK2-mediated phosphorylation of HSP27 in these processes is lacking.

Phosphorylated HSP27 was shown to participate in TNF-α-induced IL-6 synthesis in osteoblast-like MC3T3-E1 cells [111]. Overexpression of the phosphomimetic HSP27-3D mutant (in which serine residues 15, 78 and 82 are replaced by aspartic acid) resulted in higher IL-6 mRNA and protein levels than in cells overexpressing the non-phosphorylatable HSP27-3A mutant (in which serine residues 15, 78 and 82 are replaced by alanine). These results suggest a phospho-HSP27 dependent stimulation of TNF-α triggered IL-6 expression. The IL-1β-induced IL-6 release and the mRNA expression were markedly suppressed in C6 glioma cells transfected with HSP27-3D mutant, while those in the cells transfected with HSP27-3A mutant were enhanced [112]. The involvement of HSP27 in the IL-1-induced expression of IL-6 in HeLa and human fibroblast cells was also reported by another group, but the authors did not address whether the phosphorylation status of HSP27 was of importance [113]. The authors showed that HSP27 regulates the expression of IL-6 in response to IL-1 and depletion of HSP27 by siRNA reduced the transcript levels of IL-6 and COX-2 in IL-1-treated HeLa and human fibroblast cells. The inhibition of IL-6 and COX-2 mRNA was a result of instability of these mRNAs, which was caused by reduced MK2 activation. IL-1-triggered phosphorylation and activation of MK2 was inhibited by depletion of HSP27, but it is not known whether this requires phosphorylation of HSP27. Because MK2 can phosphorylate HSP27, a regulatory feedback loop can be imagined in which HSP27 mediates the activation of MK2 in response to IL-1 and activated MK2 then phosphorylates and modulates the function of HSP27. The results obtained by these different investigators indicate that phosphoHSP27 can exert opposing effects on IL-6 expression depending on the stimulus and/or cell type. In the same study, Alford and colleagues demonstrated that HSP27 is also implicated in the expression of COX-2 in response to IL-1 [113]. This confirms a report by Lasa *et al.* which reported that MK2 stabilizes COX-2 mRNA and this may involve phosphorylation of the MK2 substrate HSP27 [114]. HSP27 phosphorylation by MK2 has also been suggested to be necessary for LPS-induced IL-10 production in monocytes, but has not been unambiguously proven [115].

HSP27 can inhibit TNF-α induced activation of the NF-κB pathway [116]. The authors demonstrated that TNF-α increases the association of HSP27 with IKKβ, but not IKKα and this down-regulated NF-κB activation. Phosphorylation of HSP27 at serines 15, 78 and 82 stimulated the association of HSP27 with IKKβ. These results suggest that phosphorylated HSP27 functions as a negative regulator of the NF-κB pathway and that MK2/3/5-mediated phosphorylation may stimulate this inhibitory role of HSP27. HSP27, which is a subunit of the ARE binding protein AUF1 (AU-rich element RNA-binding protein) complex, possesses high affinity ARE-binding activity and is essential for rapid degradation of the ARE-containing TNF-α mRNA [117]. Interestingly, HSP27 in the AUF1 complex seems to be phosphorylated at Ser-82, a phosphoacceptor site for MK2/3/5, but it remains to be examined whether this phosphorylation stimulates the mRNA destabilization activity of HSP27 and whether MK2/3/5 may be implicated in HSP27-mediated TNF-α mRNA decay.

The *in vivo* importance of HSP27 phosphorylation by MK2 in inflammatory responses or diseases has not been established, but HSP27 protects against acute pancreatitis in a phosphorylation-dependent manner. Although the kinase(s) responsible for HSP27 phosphorylation was (were) not identified, these findings may point toward a putative role for MK2 [118]. 

In conclusion, unequivocal proof for a role for MK2 and phosphoHSP27 in controlling inflammatory processes is lacking. Studies with MK2 deficient mice and Hsp25 deficient mice or transgenic mice overexpressing HSP27, HSP27-3D or HSP27-3A may help shed light on the possible role of the MK2-phospoHSP27 link in inflammation. One such *in vivo* study described in the next section may provide a clue how phosphorylation of HSP27 by MK2 can modulate inflammatory responses. 

### 5.3. *In Vivo* Role of MK2 in Inflammation and Inflammatory Diseases

Using ovalbumin (OVA) challenged mice as an animal model of asthma, revealed that MK2 knockout mice were unable to mount an airway allergic inflammation [44]. Compared to wild-type mice, MK2^−/−^ mice had a reduced peribronchial inflammatory area and IL-4, IL-5, IL-10, IL-13 and IFN-γ production in lungs after immunization and exposure to OVA. Comparing TNF-α treated wild-type and MK2 deficient HUV-EC cells, the authors also showed that MK2 is essential for sustained nuclear localization of the NF-κB p65 subunit by decreasing expression of its exporter IκBα. On the other hand, MK2 is also implicated in the negative feedback of the NF-κB pathway by regulating redistribution of p38^MAPK^ from the nucleus to the cytoplasm, thereby preventing p38^MAPK^ from phosphorylating (*i.e.*, activating) nuclear MSK1. As a result, MSK1 is unable to phosphorylate p65 at Ser-276, which is required for transcriptional activation of p65 (see above). In addition, phosphorylation of HSP27 by MK2 allows phospho-HSP27 to interact with p38^MAPK^ and retain p38^MAPK^ in the cytoplasm. Thus MK2 is implicated in a complicated feedback mechanism of the NF-κB pathway involving p38^MAPK^, MSK1 and HSP27. Hence, the MK2-HSP27 pathway counteracts TNF-α-p38^MAPK^-MSK1 induced activation of NF-κB. By doing so, MK2 switches the transcriptional activity of NF-κB from high, but short lasting, to moderate, but long lasting [44]. 

The *in vivo* role of MK2 in skin inflammation was confirmed using oxazolone-induced acute allergic contact dermatitis in mice [119]. The authors showed that the inflammatory response to oxazolone is significantly reduced in MK2^−/−^ mice compared to wild-type littermates as determined by changes of ear thickness and the degree of neutrophil infiltration (monitored by myeloperoxidase activity) in the skin. In addition, MK2 deficient mice exhibit a significant reduction in TNF-α, IL-1β and IFN-γ mRNA and/or protein levels compared to wild-type mice after oxazolone challenge, which supports a pivotal role for MK2 in skin inflammation. However, studies with other skin inflammation models jeopardize the role of MK2. Decreased TNF-α, IL-1β and IL-6 expression along with myeloperoxidase activity were observed in 12-*O*-tetradecanoylphorbol-13-acetate (TPA)-induced cutaneous inflammation in MK2 deficient mice compared to treated wild-type animals, while knockout of the *mk2* gene resulted in impaired TNF-α synthesis in dinitrofluorobenzene (DNFB)- and dinitrochlorobenzene (DNCB)-driven subacute contact hypersensitivity [82,120]. However, while MK2 deficiency exerts anti-inflammatory effects in chronic TPA-induced irritative skin inflammation and in *subacute* DNFB-induced contact allergy, no inhibitory effect of MK2 deficiency on inflammation in subacute DNCB contact allergy model and in *acute* DNFB-induced contact allergy model was observed. DNFB and DNCB may differentially activate the inflammasome which may help explain the differential requirement of MK2. MK2 may be bypassed or its function is compensated for by a different kinase, such as MK3 depending on the skin inflammation model [82,121]. Cutaneous inflammation is observed in arsenic exposed humans, whereas exposing SKH-1 hairless mice to arsenic induces skin inflammation associated with increased expression of TNF-α and IL-1β and enhanced phosphorylation of MK2 in skin tissue compared to control animals [122,123]. These observations suggest, but do not prove, a role for MK2 in arsenic-induced cutaneous inflammation.

A crucial *in vivo* role for MK2 in inflammation is further supported by studies in a murine model for pancreatitis [124,125]. Proinflammatory cytokines such as TNF-α and IL-6 are involved in the pathophysiological processes of pancreatitis. Cerulein-induced pancreatitis (e.g., attenuated edema, inflammation, vacuolization, necrosis and myeloperoxidase activity) was less severe in MK2 knockout mice than in wild-type mice. Moreover, serum concentrations of TNF-α and IL-6, as well as pancreatic IL-6 transcript levels were reduced in MK2 deficient mice compared with wild-type animals. Results from these two independent studies suggest that the deletion of MK2 most likely ameliorates cerulein-induced pancreatitis in mice by decreasing the production of TNF-α and IL-6, thereby reducing inflammatory injuries caused by these cytokines. Another study revealed an opposite role of MK2 in cerulein-induced pancreatitis. Activation of the cannabinoid receptors CB1 and CB2 has been shown to mediate beneficial effects on gastrointestinal inflammations and these receptors can signal through the p38^MAPK^ and JNK pathways. Michler and co-workers wanted to explore whether the cannabinoid receptors and p38^MAPK^ or/JNK play a role in cerulean-induced pancreatitis [126]. Pretreatment with the unselective CB1/CB2 agonist HU210 improved cerulein-induced pancreatitis in wild-type and CB1 deficient mice, indicating that the beneficial effect is mediated by CB2. Indeed, stimulation with a selective CB2 agonist (JWH133) ameliorated, while a selective CB2 antagonist (AM630) deteriorated pancreatitis. Because MK2 knockout has a protective effect in cerulein-provoked pancreatitis (see above), the authors examined the effect of CB2 activation on pancreatitis in MK2^−/−^ mice. Surprisingly, they observed that knockout of MK2 abolished the protective effect of CB2 activation on cerulein-provoked pancreatitis. Thus, while disruption of MK2 or activation of CB2 does protect against cerulein-induced pancreatitis, activation of CB2 in MK2 deficient animals does not. A major difference between this study and those of Tietz and colleagues and Yi and collaborators is that Michler and colleagues modulated the activity of CB2 before they challenged the animals with cerulein. The different role of MK2 may be explained by JNK. Cerulein triggers JNK phosphorylation and in the presence of MK2, activation of CB2 did not further stimulate phosphoJNK levels, while in MK2^−/−^ animals, the CB2 agonist enhanced JNK phosphorylation. The activation state of JNK may thus affect the severity of cerulein-induced pancreatitis. Indeed, inhibition of JNK reduced the severity of cerulein-induced pancreatitis in rats [127]. The study by Mitcher *et al.* and the finding that LPS-induced JNK phosphorylation was significantly induced in MK2 siRNA-treated rat macrophages compared to scrambled siRNA transfected cells suggests a connection between MK2 and JNK [122]. The mechanism by which MK2 may impede JNK activity remains to be solved. MK5 can antagonize JNK by inhibiting the leukocyte-specific adaptor protein/hematopoietic progenitor kinase 1 (Grap2/HPK1) complex which is an activator of JNK [128]. It remains to be tested whether MK2 may operate in a similar way. Alternatively, MK2-mediated inhibition of JNK may result from decreased levels of IL-6, which is an activator of JNK [129]. IL-6 levels were strongly reduced in MK2 deficient animals [124,125,126]. It would also be interesting to monitor MK2 phosphorylation/activation in animals treated with CB2 agonists or antagonists.

Diabetic cardiomyopathy is associated with increased inflammation and is accompanied by upregulation of inflammatory modulators TNF-α, IL-1β, IL-6 and TGF-β1 [130]. Thandavarayan and colleagues had previously shown that streptozotocin-induced diabetic cardiomyopathy was accelerated in transgenic mice with cardiac-specific expression of the dominant negative 14-3-3η R56A/R60A compared to wild-type littermates, and was partly the result of enhanced Akt1 signaling [131]. The authors wanted to investigate whether perturbed 14-3-3η function also interfered with the NF-κB and p38^MAPK^-MK2 pathways. The authors observed that streptozotocin induced more phosphorylation of MK2 and NF-κB, more IκBα degradation, and a higher increase in TNF-α, IL-1β and IL-6 expression in diabetic dominant negative 14-3-3η transgenic mice compared to wild-type mice [131]. These results indicate that perturbed 14-3-3η expression exacerbates cardiac inflammation in diabetic cardiomyopathy probably by activating the p38^MAPK^/MK2 and NF-κB signaling pathways and the subsequent elevated expression of pro-inflammatory cytokines. 14-3-3 proteins have been shown to induce nuclear export of phosphorylated TTP, a RNA-destabilizing protein, resulting in increased TNF-α mRNA (see above; [87,90]). Consequently, overexpression of 14-3-3η may promote nuclear exclusion of TTP phosphorylated by MK2.

Cerebral ischemia injury, which is caused by decrease in blood supply to the brain leads to inflammation. Occlusion of the middle cerebral artery (MCAO), which causes cerebral ischemia in mice, induced expression of IL-1β in the brains of both wild-type and MK2^−/−^ mice, but IL-1β levels were significantly lower in MK2-deficient animals. These findings point to a role for MK2 in inflammation caused by cerebral ischemia [132]. As a result, inhibition of MK2 may suppress IL-1β production in the ischemic brain and might be associated with reduced ischemic damage.

Psoriasis is an inflammatory disorder of the skin. Work by the group of Iversen supports a role for MK2 in psoriasis [84]. They showed that the TNF-α protein level was significantly increased in lesional psoriatic skin compared to nonlesional skin biopsies taken from the same patients. Moreover, MK2 phosphorylation and activity were strongly increased in lesional, but not detectable in non-lesional psoriatic skin. To further elucidate the role of MK2 in inflammatory cytokine production in the epidermis, the authors used human keratinocytes. Exposure of these cells to IL-1β induced MK2 phosphorylation and TNF-α protein levels, while transfection with MK2 siRNA partially abrogated IL-1β triggered MK2 phosphorylation and TNF-α expression.

Collagen-induced arthritis in mice is a murine model used for studying human arthritis [133]. Compared to collagen-induced arthritis in wild-type mice, MK2 deficient DBA/LacJ mice did not show histological signs of arthritis and a >85% reduction in serum TNF-α and IL-6 levels was measured. The IL-6 mRNA levels correlated with the severity of the disease: Higher IL-6 mRNA levels were monitored in the paws of collagen-induced arthritis wild-type mice compared to MK2^−/−^ animals [134]. These results demonstrate the role of MK2 as a crucial player in the pathogenesis of collagen-induced arthritis, and accordingly in rheumatoid arthritis.

Studies in hypercholesterolemic mice (*ldlr*^−/−^ mice) established a causal involvement for MK2 in arteriosclerosis, a chronic inflammatory disease [135]. Development of atherosclerosis was markedly reduced in *ldlr*^−/−^/mk2^−/−^ mice *versus ldlr*^−/−^ mice after the feeding an atherogenic diet. MK2 promotes atherogenesis by stimulating the recruitment of inflammatory monocytes/macrophages and foam cell formation.

MK2 is also implicated in angiotensin II-induced vascular wall inflammation [136]. Comparative studies in wild-type and MK2 knockout mice showed that MK2 deficiency blunted angiotensin II-induced inflammatory responses such as NF-κB activation and release of the inflammatory chemokine monocyte chemoattractant protein-1 and the vascular inflammatory modulator vascular cell adhesion molecule-1. Furthermore, angiotensin-II increased both MK2 activation (phosphorylation) and MK2 expression [136].

MK2 also participates in orchestrating the expression of proinflammatory cytokines and enzymes to help control periodontal inflammation and bone loss [137]. Protein and mRNA inflammatory modulators IL-1β, IL-6, TNF-α, and COX2 were reduced by ~55–65% in MK2 siRNA-treated rat bone marrow stromal cells. *In vivo* MK2 silencing by oral administration of siRNA to rats attenuated LPS-induced inflammatory gene expression (TNF-α, IL-1β, COX2) and inflammatory cell infiltration in periodontal connective tissues. Taken together, these results confirm that MK2 siRNA delivery repressed LPS-induced inflammatory responses and that MK2 is critical in the pathogenesis of periodontitis [137]. Additionally, these studies suggest that MK2 siRNA delivery may have therapeutics applications in treatment of inflammation.

A possible contribution of MK2 in inflammatory processes in osteoarthritis was suggested by the work of Jones and colleagues who demonstrated that phosphorylated (*i.e.*, activated) MK2 is undetectable in cartilage of normal donors, while MK2 is phosphorylated in the cartilage from osteoarthritis patients [138]. Stimulation of human primary osteoarthritis chondrocytes with IL-1β triggered activation of MK2, while siRNA-mediated depletion of MK2 abrogated IL-1β-induced PGE_2_ production and strongly reduced the release of the matrix metalloproteinases 3 and 13. Hence, MK2 may participate in the deterioration of osteoarthritis. Comparative studies with bone and bone cells derived from wild-type and MK2-deficient animals play an important role in the study of bone homeostasis. Loss of MK2 may affect bone health and deteriorate chronic inflammatory diseases such as rheumatoid arthritis and ankylosing spondylitis which can directly cause osteoporosis [139]. Results from both studies indicate that MK2 may be a therapeutic target in certain bone pathologies.

MK2 is implicated in the pathogenesis of inflammatory bowel disease, a group of chronic intestinal inflammatory diseases that include ulcerative colitis and Crohn’s disease. Inflammatory bowel disease is characterized by the perpetual production of inflammatory mediators such as IL-1, IL-6, TNF-α and INF-γ. The p38^MAPK^-MK2 pathway plays an important role in the production of these molecules, which participate in intestinal inflammation [140].

Finally, both MK2 expression and activity are induced in *in vivo* models of neuroinflammation, which may guarantee optimal production of pro-inflammatory mediators [80]. A study by Culbert and co-workers demonstrated that MK2 is also a key actor in the persistent neuroinflammation observed in brains from Alzheimer disease patients [79]. It has been proposed that the elevated levels of β-amyloid protein (Aβ), a pathological hallmark for of Alzheimer disease, induce microglia to release pro-inflammatory mediators leading to microglial autoactivation and amplification the inflammatory response. This neuroinflammation is assumed to contribute to Alzheimer disease. *In vitro* studies with wild-type and MK2 deficient microglia demonstrated that release of TNF-α, keratinocyte chemoattractant (KC, the murine equivalent of human IL-8), and macrophage inflammatory protein 1α (MIP-1α) was significantly reduced in MK2^−/−^ miroglia cells compared to wild-type cells after treatment with LPS+IFN-γ, while MIP-1α secretion was significantly reduced after exposure to Aβ peptide. LPS+IFN-γ or Aβ-stimulated microglia caused cell death of co-cultured cortical neurons, which was abolished when using MK2 deficient microglia. Using a mouse model of Alzheimer disease (*i.e.*, double transgenic mice that overexpress mutant forms of human amyloid precursor protein and presenilin-1 specifically in the CNS), the authors could show that TNF-α, MIP1-α, and KC that MK2, TNF-α, MIP1-α, and KC expression was significantly up-regulated in the cortex but not the cerebellum. Microglia were activated and MK2 activity was increased compared with wild-type animals. These results suggest that MK2 is pivotal in regulating chronic inflammation in the brains of Alzheimer disease patients and makes MK2 an attractive therapeutic target. Reduced LPS-induced inflammation was also observed in MK2-deficient neuron-glia cultures and mice [80]. The authors observed that the production of TNF-α and IL-6 by MK2^−/−^ mesencephalic neuron-glia cultures in response to LPS was strongly reduced (–85% and –75%, respectively) compared to wild-type cultures. A three-fold induction of TNF-α synthesis was measured in wild-type mice treated with MPTP (=1-methyl-4-phenyl-1,2,3,6-tetrahydropryidine), a neurotoxin used to induce Parkinson’s disease. However, MPTP failed to trigger TNF-α expression in MK2^−/−^ mice [80]. MK2 also participates in the inflammatory response after spinal cord injury [141]. Microarray assays using spinal cord RNA prepared 7 days after spinal cord injury revealed that MK2 mRNA was one of the transcripts that was up-regulated (1.7-fold) in MK2^−/−^ mice relative to wild-type controls. MK2 and phospho-MK2 protein levels increased after injury, peaking at 7 days postinjury. Twelve hours after spinal cord injury, phospho-MK2 was primarily detected in neurons and astrocytes, while 7 days postinjury, the number of phospho-MK2 positive cells was strongly reduced in astrocytes, but increased in neurons and microglia. Early after injury, *mapkapk2 null* mice showed reduced expression of the pro-inflammatory cytokines TNF-α (26.5-fold) and IL-12 (2.6-fold) in the spinal cord, while hardly any neutrophil influx had occurred in the injured spinal cord of MK2^−/−^ mice. No differences in the number of macrophages in the injured spinal cord between wild-type and MK2^−/−^ mice were observed. Early after injury, neurons may be the cells producing the pro-inflammatory cytokines, while at a later time microglia, which are resident macrophages in the brain, may also contribute to the production of pro-inflammatory cytokines. Taken together, these data suggest that activated MK2 plays a role in triggering spinal cord injury-induced inflammation, while MK2 deficiency reduces inflammatory responses. 

## 6. MK3 and Inflammation

MK3 is the MAPKAPK that is most structurally related to MK2 and is also directly activated by p38^MAPK^ [13]. In contrast to MK2-deficient mice, LPS-challenged MK3 knockout animals did not display a change in the mRNA stability of inflammatory modulators such as IL-1, IL-8, IL-10, IL-12, IFN-γ and TNF-α compared to wild-type mice [15]. The authors speculated that the differences between MK2- and MK3-deficient mice may result from differences in MK2 and MK3 expression levels and activity because MK2 is more abundantly expressed than MK3, whereas MK2 activity is more prominent than MK3. Hence, the dominancy of MK2 may mimic the phenotypical changes in MK3-deficient mice and MK2/MK3 double knockout cells or mice are therefore often used to elucidate the biological role of MK3. LPS-induced TNF-α biosynthesis in MK2/MK3-deficient BMDM and animals was further reduced compared to LPS-treated MK2^−/− ^BMDM and mice. Moreover, ectopic expression of MK3 stabilized mRNA levels of several LPS-induced genes. Interestingly, MK3 was also reported to phosphorylate hnRPN A0 (see above)*in vitro* at Ser-84. As outlined above, MK2-mediated phosphorylation of hnRNP A0 at Ser-84 is assumed to stimulate translation of TNF-α mRNA [86]. In accordance, MK3 may regulate TNF-α production and stabilization of mRNA for inflammatory regulators in response to LPS, which suggests that MK3 is implicated in inflammatory responses. 

A recent study has revealed that MK2 and MK3 fulfill opposite effects in the regulation of LPS-induced IFNβ expression in macrophages [142]. STAT3 is a major mediator of anti-inflammatory effects in macrophages, while MK2 is important for delayed STAT3 activation in response to LPS because it controls the synthesis of IFNβ, a cytokine that can activate STAT3 [142,143]. LPS-induced IFNβ mRNA levels were substantially reduced in MK2^−/−^ macrophages compared to wild-type cells. Surprisingly, IFNβ levels in MK2/MK3 double knockout macrophages were higher than in wild-type cells, thereby suggesting that MK3 exerts negative effects on IFNβ expression which are counteracted by MK2. The reduction in IFNβ transcripts in MK2-deficient macrophages was not the result of increased mRNA decay, but the authors showed that MK2 regulates LPS-induced expression of IFNβ at the transcription level. To test this, the authors investigated the effect of MK2 and MK3 on interferon regulatory factor 3 (IRF3) and NF-κB, both of which are essential transcription factors in IFNβ gene transcription. They found that MK2 mediates the LPS-induced phosphorylation of IRF3 at Ser-396, which is necessary for activation, whereas MK3 exerts negative effects on IRF3 function. In addition, MK2 prevents MK3 from impeding nuclear localization of the NF-κB p65 subunit. MK2 assures an accurate NF-κB nuclear translocation by controlling basal protein levels of IκBβ, a protein that transports p65 out of the nucleus, and by preventing MK3 from impeding its degradation. This fine-tuned mechanism allows MK2 to tightly control the induction, propagation and resolution of the inflammatory macrophage response [83]. Reduced IL-10 production by MK2^−/−^ BMDM was not further affected in MK2/3^−/−^ BMDM, thus indicating that MK3 does not exert a negative role on MK2-regulated IL-10 expression [142]. 

## 7. MK5 and Inflammation

Convincing evidence for the contribution of MK5 in inflammatory responses is lacking. In contrast to MK2 knockout mice, MK5^−/−^ mice possess the same susceptibility to endotoxic shock as wild-type mice and no significant difference in the biosynthesis of TNF-α, IL-6, or IFN-γ in spleen cells from MK5-deficient mice has been observed compared to MK5^+/+^ spleen cells after LPS stimulation. In MK2/MK5 double knockout animals, the LPS-induced production of TNF-α is similar to levels measured in MK2^−/−^ mice [144]. Taken together, these results suggest redundancy of MK5 in LPS-induced inflammatory responses. 

MK5 phosphorylates *in vitro* two proteins implicated in inflammatory responses: cPLA_2_ at Ser-727 and hnRNP A0 at Ser-84, although the *in vivo* phosphorylation is not clear and biological significances in inflammation are not known [67,86]. HSP27 is also a genuine substrate for MK5, but MK5 seems to phosphorylate HSP27 in response to activation of the cAMP/protein kinase A pathway, rather than stress [110]. There is no indication so far that the MK5/HSP27 connection is implicated in inflammation.

**Figure 2:**
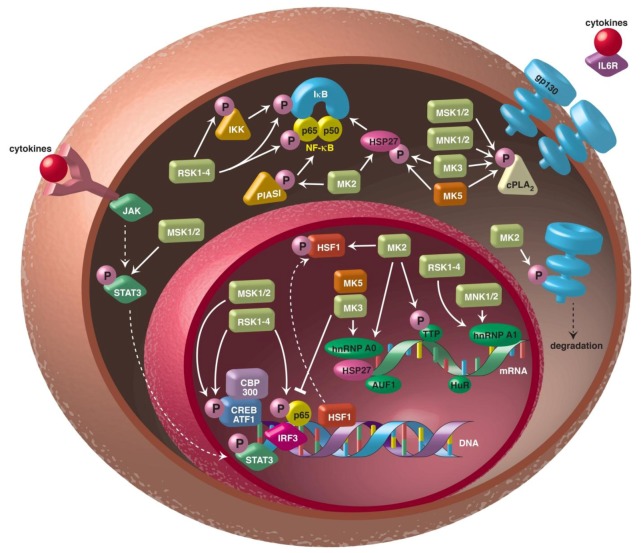
MAPKAPK can interfere with inflammatory processes by different mechanisms. MAPKAPKs can control signalling pathways such as the nuclear factor-kappa B (NK-κB), JAK/STAT3 and IL6R/gp130 pathways. They can also affect biosynthesis of inflammatory modulators at the transcriptional level by modulating the activity of transcriptional activators (e.g., CREB, IRF3, p65, CBP/p300) or repressors (e.g., HSF1). MAPKAPK can also regulate the stability of transcripts by controlling the activity of mRNA binding proteins such as HuR, TTP, AUF1, hnRNP A0 and hnRNP A1. The MAPKAPK MAPKAPK-3 (MK3), MAPK-interacting kinases (MNK), the mitogen- and stress-activated kinases (MSK) and MK5 may also stimulate the enzymatic activity of cytoplasmic phospholipase A2 (cPLA2). Abbreviations: ATF1, activating transcription factor-1; AUF1, AU-rich element RNA-binding protein 1; CBP, CREB-binding protein; cPLA2, cytosolic phospholipase A2; CREB, cAMP response element-binding protein; gp130, glycoprotein 130; hnRNP, heterogeneous nuclear ribonucleoprotein; HSF1, heat-shock factor 1; HSP27, heat-shock protein 27; HuR, human antigen R; IKK, I kappa B kinase; IL6R, interleukin-6 receptor; IRF3, interferon regulatory factor 3; JAK, Janus kinase; p300, histone acetyltransferase; PIAS1, protein inhibitor of activated STAT1; STAT, signal transducer and activator of transcription; TTP, tristetraprolin. See text for details.

The involvement of the different MAPKAPKs in inflammatory processes and diseases is summarized in Figure 2.

## 8. MAPKAPK Inhibitors in the Treatment of Inflammatory Diseases

Chronic inflammation is a hallmark in several disorders, amongst others atherosclerosis, autoimmune diseases, neurodegenerative diseases, and allergies. The development of good anti-inflammatory drugs is therefore important. Traditional treatment includes glucocorticoids, non-steroidal anti-inflammatory drugs, TNF-α antibodies, cytostatic drugs [145,146]. However, most of these drugs are expensive, have side effects and not all patients have a positive response. Because the central role of MAPK pathways in inflammation became obvious, a lot of efforts have been spent to develop specific drugs against components of the pathways in the hope that they can be used to treat chronic inflammation. Several MAPKAPK inhibitors have been designed, but few have entered the clinic.

### 8.1. RSK Inhibitors

The first specific RSKs inhibitors were the kaempferol glycoside SL0101 (3″,4″-di-O-acetyl-α-l-rhamnopyranoside) and a fluoromethylketone inhibitor (FMK), followed by the development of more potent derivatives with higher target specificity [147,148,149,150,151,152,153]. So far, none of these RSK inhibitors have been tested in clinical trials with patients suffering from inflammatory diseases.

### 8.2. MSK1/2 Inhibitors

Despite their off-target inhibition of several other protein kinases, H-89 and Ro 31-8220 have been used in several biochemical studies to inhibit MSK1/2 [154]. Recently, the novel MSK1/2 inhibitor SB-747651A with an IC_50_ = 11 nM and improved specificity has been developed [155,156]. Against a panel of >100 protein kinases, SB-747651A reduced the activity of four other kinases, including RSK1, to a degree similar to MSK1. SB-747651A impairs the production of IL-10 in macrophages following LPS exposure. Similar to the increased secretion of TNF-α in MSK1/2 knockout in response to LPS (see above), SB-747651 treatment increased TNF-α secretion in LPS-challenged BMDM [157]. Glucocorticoids are anti-inflammatory agents that provoke the subcellular distribution of MSK1 to the cytoplasm, thereby counteracting MSK1 recruitment to inflammatory gene promoters [158]. As such, glucocorticoids can function as rather unspecific MSK inhibitors. Glucocorticoids have been used to treat patients with inflammatory diseases, but their use can lead to resistance or insensitivity [159].

### 8.3. MNK Inhibitors

The MNK inhibitor CGP57380 (4-amino-3-(*p*-fluorophenylamino)pyrazolo [3,4-d] pyrimidine) was identified from the Novartis Pharma compound collection by *in vitro* kinase assays [160]. CGP57380 impaired Shiga toxin-, LPS- or anisomycin-induced biosynthesis of cytokines in macrophages and/or keratinocytes, but has not entered clinical trials [74,75,161,162].

### 8.4. MK2/3 Inhibitors

Because studies in cell culture and animal models have confirmed the pivotal role of MK2 in inflammation, most research has focused on developing specific MK2 inhibitors. However, targeting MK2 may lead to a compensatory increase in MK3 activity given that MK2 and MK3 have overlapping functions and that the overexpression of MK3 can rescue the phenotype of MK2-deficient mice which may unmask the properties of MK3 as outlined in Section 6 [15]. PF-3644022 [(10*R*)-10-methyl-3-(6-methylpyridin-3-yl)-9,10,11,12-tetrahydro-8*H* [1,4] diazepino [5’,6’: 4,5] thieno [3,2-f] quinolin-8-one] is a benzothiothiophene type specific MK2 inhibitor with a IC_50_ of –5 nM (5 nM for MK5 and 53 nM for MK3, while the IC_50_ for the other MAPKAPKs is 30- to 600-fold higher compared to MK2). It exhibits a high specificity when assayed against a diverse panel of 200 human protein kinases. PF-3644022 is orally efficacious in suppressing TNF-α production in acute LPS challenged rats and in chronic inflammation in streptococcal cell wall-induced arthritis in rats [163]. However, the biochemical efficiency of PF-3644022, the ratio of binding affinity to target *versus* cellular activity is low (0.03) compared to most drugs on the market and the hepatotoxicity observed in dogs and monkeys necessitates the search for better MK2 inhibitors [91]. Screening a library of –100,000 molecules for MK2 inhibitory activity identified a pyrazol[1,5-a] pyrimidine compound with a MK2 IC_50_ = 1.3 μM. Modifications resulted in Compound 44 and 64 with an IC_50_ of 0.40 μM and 0.054 μM , respectively. The protein kinase selectivity of Compound 44 was evaluated against >100 kinases and the majority of kinases was inhibited by less than 30%, while five kinases (MK2, CHK2, Yes, Fes, Flt3) were inhibited by >75%, with MK2 being the most inhibited. Compound 44 impaired LPS-induced TNF-α production and HSP27 phosphorylation in a concentration-dependent manner *in vitro*, while this compound also reduced serum TNF-α levels in mice intraperitonally injected with LPS, though clinical trials have not been started [164]. Several other selective MK2 inhibitors with an IC_50_ in the nM range and the ability to inhibit LPS-induced TNF-α production in cell cultures or animals have been developed [165,166,167,168,169,170].

Peptide MK2 inhibitors have also been developed [171,172,173]. LPS-treated THP-1 human monocytes with the MK2 inhibitor peptide KALARQLGVAA covalently linked to cell-penetrating peptides significantly reduced TNF-α, IL-6 and IL-8 production [173]. Other investigators have reported a lack of IL-8 downregulation by a competitive MK2 inhibitor peptide, which can probably be attributed to the poor specificity of the MK2 inhibitor peptide [171]. The MK2 peptide inhibitor MMI-0100 inhibited TNF-α provoked IL-6 secretion by endothelial cells [174]. 

Specific MK3 inhibitors have not yet been identified, though some MK2 inhibitors also act against MK3 with comparable IC_50_ (see section above). The MK5 inhibitor noroxoaconite also inhibited MK3, but not MK2, making this compound a potential MK3 therapeutic drug in inflammatory diseases [175]. However, the effect of this inhibitor on inflammatory processes has not been tested.

### 8.5. MK5 Inhibitors

Recently, several specific MK5 inhibitors have been reported, however few of them have been assayed for anti-inflammatory properties in animal models or have entered clinical trials [176,177,178,179,180]. GLPG0259 reduced inflammation in the mouse collagen-induced arthritis model and was safe and well-tolerated in healthy subjects [176,179]. However, when used in a phase II study on patients with active rheumatoid arthritis (RA), this drug did not display any signs of efficacy. This may indicate that MK5 is not implicated in the pathogenesis of RA or that the blocking of MK5 may lead to compensatory effects of other pathways [180].

## 9. Conclusions

Although a role for different MAPKAPKs in controlling inflammatory responses is emerging, MK2 seems to be the most central member in these processes. Studies with MK2 deficient mice and cells have been pivotal in scrutinizing the mechanisms by which this kinase participates in inflammation and inflammatory diseases. MK3, which is most closely related to MK2, may imitate some inflammatory functions of MK2, but intriguingly this protein also exerts opposite effects under certain conditions. RSK can modulate expression levels of inflammatory modulators at transcriptional and post-transcriptional levels, whereas MNK predominantly controls stability of mRNAs encoding inflammatory proteins. The use of single or double MNK1/2 knockout mice may be a helpful tool in further exploring the contribution of these MAPKAPKs in inflammation. The NF-κB pathway fulfils a central role in several inflammatory responses and seems to be targeted by several MAPKAPKs, including RSK, MK2 and MSK1. However, the mechanism by which MSK1 affects activity deserves further attention because the suggested activation of the p65 subunit of NF-κB by MSK1-mediated phosphorylation at Ser-276 may be a technical pitfall [47]. Moreover, this site is an autophosphorylation site, jeopardizing the role of MSK1 as a genuine p65 kinase. Studies with e.g., knock-in mice expressing p65 S276A may be useful in solving the possible MSK1:p65 link in inflammation, but unfortunately these mice die at different embryonic days, making studies aimed at the physiological function of phosphoSer-276 p65 difficult [181]. Although MK5 is also activated by p38^MAPK^, no obvious role in inflammation has been discovered so far. Complete sequencing of genomes and transcripts from patients with different inflammatory diseases and meticulous studies of the inflammasome may disclose novel roles for MAPKAPKs in inflammation. The pivotal functions of MAPKAPKs and especially MK2 in inflammatory diseases make these protein kinases attractive targets for the development of therapeutic strategies. However, because MAPKAPKs control pro-inflammatory, as well as anti-inflammatory processes, inhibition may enhance suppression and decrease amplification of the inflammatory response, hence resulting in an unwanted effect of the therapy. Inflammatory pathways also promote tumour development, hence drugs against MAPKAPKs may not only be beneficial for inflammatory disorders, but for malignant diseases as well as.
